# Assessment of Elementary School Teachers' Level of Knowledge and Attitude regarding Traumatic Dental Injuries in the United Arab Emirates

**DOI:** 10.1155/2017/1025324

**Published:** 2017-09-14

**Authors:** Manal A. Awad, Eman AlHammadi, Mariam Malalla, Zainab Maklai, Aisha Tariq, Badria Al-Ali, Alaa Al Jameel, Hisham El Batawi

**Affiliations:** ^1^Department of Preventive and Restorative Dentistry, College of Dental Medicine, University of Sharjah, Sharjah, UAE; ^2^Dubai Health Authority, Dubai, UAE; ^3^Princess Nourah Bint Abdulrahman University, Riyadh, Saudi Arabia

## Abstract

**Introduction:**

In this cross-sectional study, the level of knowledge and attitude of elementary school teachers regarding traumatic dental injuries (TDI) were assessed.

**Materials and Methods:**

A questionnaire was distributed to 330 elementary school teachers in 30 randomly selected schools in the Emirates of Sharjah and Dubai. The questionnaire collected information on participants' demographic characteristics, first aid training, and attitude about emergency management of TDI.

**Results:**

292 teachers (88%) completed the questionnaires; of these, 95% were females, and 50% of the participants had first aid training. Knowledge about tooth avulsion was inadequate, and first aid training was not associated with correct responses to management of avulsed teeth (*p* > 0.05). A significantly higher percentage of younger teachers (*p* < 0.05) expressed the need for future education on TDI management. A significantly higher percentage of participants who had an educational position (95%) indicated that they did not have enough knowledge regarding TDI compared to physical education teachers (79%) and administrators (87%) (*p* < 0.05).

**Conclusions:**

Elementary school teachers in the UAE have a low level of knowledge regarding the management of dental trauma. Educational programs that address TDI are needed and could improve the elementary school teachers' level of knowledge in emergency management of TDI.

## 1. Introduction

Traumatic dental injuries (TDI) among children are considered a public health concern [1] Dental injuries can lead to tooth loss; subsequently it can have a negative impact on children's psychological well-being [2–4]. A study from Brazil showed that adolescents with severe untreated TDI were 2.4 times more likely to report worse oral-health-related quality of life than adolescents without untreated TDI [4]. Accordingly, the prognosis of injured teeth depends on immediate and appropriate management by those present at the site where the trauma took place; this includes schoolteachers and staff [4, 5].

Studies have demonstrated that TDI are sometimes not properly treated, one of the reasons for this problem being the time between the trauma episode and seeking dental treatment, which can sometimes be years [6].

The main cause of TDI among school-age children are unpreventable falls in schools. It has been previously reported that approximately 50% of schoolchildren have experienced a TDI prior to graduation [7]. Therefore, it is highly likely that first aid would be provided by teachers and other school staff. Accordingly, teachers' knowledge of the management of TDI is essential to improve prognosis. Although many studies have been conducted in different countries to assess schoolteachers' knowledge of TDI [1, 2, 6–11], only one has previously been conducted in the UAE [12], and this included only Arabic-speaking teachers in the Emirate of Ajman.

The main objective of this study is to evaluate the level of knowledge and attitudes of elementary schoolteachers in two Emirates in the UAE, Dubai and Sharjah, regarding TDI among school-age children.

## 2. Material and Methods

In this cross-sectional study, a self-administered questionnaire was distributed to 330 elementary school teachers in the Emirates of Sharjah and Dubai. Permission to conduct the study was obtained from the educational authorities of the participating schools. Schools principals approved participation of the school teachers in the study and all participating teachers signed consent forms. The questionnaire used in this study is based on those previously used in Jordan [6] Saudi Arabia [8], Australia [13], and Iran [9]. This questionnaire consisted of four parts. Part 1 included questions related to the demographic characteristics of the study participants, including gender, age, position, first aid training, and experience with dental trauma. Part 2 included questions related to participants' attitude towards emergency management of dental trauma. These attitude questions included 10 items with five possible answers for each (strongly agree, agree, disagree, neither agree nor disagree, agree, and strongly disagree). Part 3 provided participants with two case scenarios of dental injuries. The first concerned a fractured tooth in a nine year old, and the second was a case of a 13-year-old child with an avulsed tooth. These two scenarios had previously been used to assess schoolteachers' knowledge about dental injuries in other studies [5, 12]. For each scenario, participants were given several options to choose from. Part 4 of the questionnaire was related to self-assessment, in which participants were asked the following three questions: (a) “Is your knowledge on dental emergency management enough?” (b) “Do you need future education in this regard?” (c) “Are you able to provide proper action when needed?” For each question, participants gave a yes or no answer.

The questionnaire was translated to Arabic and backward and forward translation was used to establish equivalency. Because some teachers were not Arabic-speaking, participants were given the option of responding either to the English or the Arabic version of the questionnaire.

### 2.1. Statistical Analysis

Data were analyzed using the statistical analysis package SPSS (Version 20, Chicago, IL). Results were analyzed by frequency distribution. Chi-square tests were also utilized to assess the effect of first aid training on knowledge of management of presented cases. In addition, Chi-square tests were also used to evaluate the relationship between self-assessments of knowledge, need for additional education, and proper action according to gender, position, school, age, and first aid training. For all assessments, level of significance was set at alpha 0.05, two-tailed.

## 3. Results

Of the 330 teachers who were approached from 30 randomly selected schools in Sharjah and Dubai, 88%  (*N* = 292) responded to the questionnaire. The majority of participants were females (*N* = 278; 95%) and approximately half of school teachers had first aid training (*n* = 146; 51%) (Table 1). In this study, 36% of the participants indicated that teachers are not responsible for traumatic dental injuries, and the majority (89%) believed that time is an important factor in the prognosis of dental trauma. In addition, 81% indicated that educational experience can assist in the management of TDI (Table 2).

Table 3 depicts the participants' responses to the two cases presented to them. Only 58% knew that the damaged tooth was permanent. More than half of the participants (*n* = 165; 57%) responded that the immediate action would be to contact the parents and advise them to send the child to the dentist. Only 33% knew that the correct immediate action is to look for the broken tooth and send the child to the dentist.

Table 3 also summarizes the responses to questions regarding tooth avulsion. Only 6% selected the option to wash the tooth and put it back in its place in the mouth, while the majority (*n* = 174; 61%) thought that stopping the bleeding by compressing a cloth over the injury was the correct immediate action. Of all the participants, 12% indicated that they would go to a general dentist. When asked about tetanus vaccination, more than half (*n* = 163; 59%) responded correctly; 31% did not know what to do if the tooth fell on dirty ground, and 32% gave the correct answer that the tooth should be rinsed under tap water and put back into the socket. Forty-six percent of the participants thought that the tooth should be wrapped in a handkerchief or paper tissue to be transported to the dentist. The highest percentage (41%) thought that the liquid that the tooth should be stored in is tap water. Only 23% indicated that the best time for the tooth to be replaced in its socket was immediately after the accident.

Table 4 shows that first aid training did not make a significant difference to the correctness of participants' responses.

A significantly higher percentage of younger participants (<35) acknowledged that they needed future education on dental emergency management compared to other groups (*p* < 0.05) (Table 5). In addition, 94% of those with an educational position did not have enough knowledge of management of dental emergencies compared to other groups (*p* < 0.05). In addition, a significantly higher percentage of teachers from government schools (93%) believed that they could benefit from further education on TDI compared to those in private schools (78%). First aid training was not related to self-assessment regarding dental emergency management.

## 4. Discussion

This study included teachers from randomly selected schools in the Emirates of Sharjah and Dubai. Similar to previous reports from Iran [12], Jordan [6], and Brazil [2], approximately half of the teachers (51%) in this study had first aid training. However, this percentage is higher than previously reported in the UAE [12], in which only 32% of school teachers in the Emirate of Ajman had first aid training and in Saudi Arabia, in which only 18% of primary school teachers had first aid training [8]. As previously reported [9], this training did not contribute to providing correct answer to the case scenarios provided. These findings highlight the importance of continuing education programs that are specifically directed towards management of TDI. Moreover, in this study, 81% of the school teachers indicated that short educational programs could assist them in the management of TDI. More than half of the teachers agreed that they are responsible for the management of dental trauma when it occurs in schools. These findings emphasis the need for tailored educational activities.

Consistent with a previous study in the UAE [12], more than half of the participants (58%) in this study did not know that the broken tooth in the nine-year-old child was a permanent tooth. Lack of knowledge about the eruption timing of teeth suggests that teachers may take actions that could have future negative consequences. For example, it is important that teachers and school staff are aware that an avulsed primary tooth should not be reimplanted to avoid/reduce damage to succeeding permanent tooth, while an avulsed permanent tooth should be immediately reimplanted to keep periodontal cells viable [14, 15].

In case 2, few (23%) participants responded that an avulsed tooth must be replanted immediately and the highest percentage of teachers (43%) did not know what to do. Similar results were observed in studies in Iran [7] and the UAE [12], in which 39% and 19%, respectively, of participating teachers indicated that they did not know what to do regarding the appropriate time to replant an avulsed tooth. Although immediate implantation of an avulsed tooth is necessary for long-term prognosis and reduction of possible complications, an obstacle for immediate action is the lack of parental consent [12, 16].

The lack of knowledge among elementary school teachers about TDI calls for action to improve education about the types of teeth that are likely to suffer from TDI and the proper manipulation and handling of teeth to maintain vitality of cells. Moreover, our findings show that 30% of teachers had witnessed TDI; this indicates that TDI are not uncommon and that schools in collaboration with health authorities should take action to educate teachers about proper management of TDI. Furthermore, reports from previous studies [17–20] showed that providing teachers with information that specifically addressed teeth avulsion had enhanced their knowledge about TDI. For example, Pujita et al. [17] examined primary school teachers' knowledge on TDI before and after an intervention, in which a promotion program was implemented on TDI. The authors reported that teachers' knowledge significantly improved as a result of providing them with the promotion sessions. In another study, Lieger et al. [19] tested the effect of posters displayed in schools on teachers' management of TDI. Findings from this study showed that, five years after the poster campaign, teachers who worked in the areas where the posters were displayed had significantly better knowledge in management of TDI compared to teachers who worked in other areas. These findings are encouraging and could be easily adopted, and their effects could be examined in elementary schools in the UAE as well.

Approximately 37% of the teachers in this study reported that they could provide proper action in case of TDI, and compared to other groups, a significantly higher percentage of younger teachers (<35 years old) indicated that they were not able to provide proper action in this regard. These findings suggest that teachers in general may have overestimated their knowledge, which may lead to mismanagement of broken or avulsed teeth in children. However, younger teachers are more likely to admit to their lack of ability to handle TDI.

Recently, Al-Musawi et al. [21] demonstrated that providing information on the management of TDI through the use of smartphone applications among a group of 28 schoolteachers was significantly more effective than providing lectures only (*N* = 32) on management of avulsed teeth. The use of such a device is worth exploring in a more pragmatic situation in which stress is an important factor that should also be taken into consideration.

## 5. Conclusion

The results of this study show that there is a need for education about TDI among school teachers in the UAE. Our findings also highlight the fact that more than one-third of the teachers and staff did not think that handling TDI was their responsibility. These disturbing findings should be addressed and teachers should be informed about the important role that they could play in handling TDI, as well as the negative consequences of poor management or neglect of TDI.

Educational programs and training are needed to improve proper management of traumatic injuries by schoolteachers and avoid unnecessary negative consequences. Guidelines on the management of TDI in schoolteachers' training curriculum should also be given serious consideration.

## Figures and Tables

**Table 1:** Characteristics of study participants.

Variable	*N* (%)
Gender	
Males	14 (4.8)
Females	278 (95.2)
Age group	
<35	148 (51)
36–45	102 (35)
>45	42 (14)
Education	
High school/diploma	58 (20)
Bachelor, masters	227 (80)
Position	
Educational	246 (87)
Health/physical education	14 (5)
Administration	24 (9)
First aid	
Yes	146 (51)
No	49 (49)
Dental emergency training	
Yes	13 (5)
No	249 (95)
Witness	
Yes	84 (29)
no	201 (71)

**Table 2:** Participants attitude towards traumatic dental injuries.

Questions	Strongly agree/agree
	*N* (%)
A teacher is not responsible for posttraumatic dental injuries	104 (36)
Time consciousness for emergency management of dental trauma can play a vital role in improving tooth prognosis	282 (89)
A tooth after avulsion will be lost definitely, so there is no need for treatment	35 (12)
Dental trauma emergency management must become one of the educational priorities for teachers	177 (61)
Dental trauma emergency is not an emergency situation	41 (14)
Teacher intervention in school dental injuries may play a key role in traumatized tooth	212 (73)
Emergency management of dental trauma requires special education and therefore there is no need for teacher intervention	114 (40)
Wearing a mouth guard should be compulsory in all contact sports	186 (65)
Due to legal considerations, it is advisable that a teacher refrains from intervening in such scenarios	102 (35)
Having some short pertinent educational experiences, educators can provide better assistance in traumatic dental scenarios	232 (81)

**Table 3:** Teachers responses to two scenarios on traumatic dental injuries.

Scenario	*N* (%)
*Case 1*	
During school hours, a 9-year-old child is hit in the face with softball. Her upper front tooth is broken. Otherwise, she is healthy, unhurt, and conscious The broken tooth is likely to be	
(i) Temporary	73 (25)
(ii) Permanent	166 (58)
(iii) Do not know	49 (17)
Your immediate emergency management of the case is	
(i) Calm down the child and send her back to class	6 (17)
(ii) Contact parents and advise them to send child to the dentist immediately	165 (57)
(iii) Look for broken tooth piece and send child to the dentists with it	96 (33)
(iv) Do not know what to do	10 (4)

*Case 2*	
A 13-year-old boy is hit in the face and his upper front tooth is missing and there is blood in his mouth. Otherwise, he is unhurt, healthy, and he did not lose consciousness	
The immediate emergency action you would take is	
(i) Stop the bleeding by compressing a cloth over the injury	174 (61)
(ii) Look for the tooth, wash it, and put it back in its place	17 (6)
(iii) Save the tooth in child's mouth and look for professional help	53 (18)
(iv) Place the tooth in a paper and send the child to dentist after the school time	26 (9)
(v) Do not know what to do	14 (5)
What type of health services would you seek first?	
(i) General physician	34 (11)
(ii) Pediatric physician	13 (5)
(iii) Hospital	27 (10)
(iv) Dental School University	11 (4)
(v) General dentists	97 (33)
(vi) Pediatric dentist	96 (33)
(vii) Endodontist	7 (2)
Would you investigate if the child had tetanus?	
(i) Yes	163 (59)
(ii) No	113 (41)
If the tooth has fallen on the dirty ground what would you do?	
(i) Rinse the tooth under tap water and put it back into its socket	90 (32)
(ii) Rub away the dirt by a sponge and soap and put it back	17 (6)
(iii) Put it back into the socket immediately without cleaning	5 (2)
(iv) Discard the tooth	81 (29)
(v) Do not know what to do	87 (31)
How would you transport the tooth on the way to the dentist if you cannot put the tooth back into its socket?	
(i) Put the tooth in ice	58 (20)
(ii) Put the tooth in liquid	76 (27)
(iii) Place the tooth in the child's mouth	19 (7)
(iv) Place the tooth in the child's hand	2 (1)
(v) Wrap the tooth in a handkerchief or paper tissue	130 (46)
Mark desirable liquids for storing a tooth that has been knocked out while you are on your way to the dentist	
(i) Tap water	100 (37)
(ii) Fresh milk	23 (9)
(iii) Child's saliva	29 (11)
(iv) Alcohol	9 (3)
(v) Saline solution	65 (24)
(vi) Disinfecting solution	44 (16)
(vii) Chicken egg white	0 (0)
Which is the best time for putting back a tooth in if it is knocked out of the mouth?	
(i) Immediately after the accident?	66 (23)
(ii) Within 30 min after the bleeding has stopped	55 (19)
(iii) Within the same day	18 (6)
(iv) This is not a crucial factor	23 (8)
(v) Do not know what to do	122 (43)

**Table 4:** Relationship between first aid training and teachers responses to the case scenarios^a^.

Questions	First aid course		*p* value
	Yes	No	
(1) Emergency management			*p* = 0.2
Correct	89 (62)	77 (55)	
Other responses	55 (38)	64 (45)	
(2) The best health services			
Correct	51 (35)	45 (32)	*p* = 0.50
Other responses	92 (65)	97 (68)	
(3) Tetanus vaccine			*p* = 0.83
Correct	9 (6)	8 (6)	
Other responses	133 (94)	131 (94)	
(4) Cleaning before replantation of a dirty tooth	53 (38)	42 (30)	*p* = 0.37
	87 (62)	98 (70)	
(5) Transportation vehicle	48 (35)	42 (30)	*p* = 0.37
	89 (65)	98 (70)	
(6) Storage media			
Correct	116 (81)	106 (76)	*p* = 0.31
Incorrect	27 (19)	33 (24)	
(7) Time to replant avulsed tooth			*p* = 0.11
Correct	39 (51)	103 (48)	
Incorrect	27 (41)	113 (52)	

^a^Based on Chi-square test.

**Table 5:** Relationship between self-assessment and sociodemographic factors and first aid training^a^.

Variables	Knowledge		Need future education		Proper action	
	Enough	Not enough	Yes	No	Yes	No
	*N* (%)	*N* (%)	*N* (%)	*N* (%)	*N* (%)	*N* (%)
Gender						
Male	3 (21)	11 (79)^b^	11 (79)	3 (21)	5 (36)	9 (64)
Female	19 (7)	254 (93)	218 (80)	53 (20)	100 (37)	166 (63)
Age						
<35	10 (7)	136 (93)	111 (77)	34 (23)	46 (32)	97 (68)^b^
36–45	6 (6)	95 (94)	86 (85)	15 (15)	39 (39)	60 (61)
>45	69 (15)	34 (85)	32 (82)	7 (18)	20 (19)	18 (11)
Position						
Educator	14 (6)	228 (94)^b^	193 (80)	47 (20)	84 (36)	152 (64)
Health teacher	3 (21)	11 (79)	14 (100)	0 (0)	7 (54)	6 (46)
Administration	3 (13)	21 (87)	20 (83)	4 (17)	9 (38)	15 (63)
First aid						
Yes	11 (8)	131 (92)	116 (82)	26 (18)	51 (37)	88 (63)
No	11 (8)	131 (92)	111 (79)	29 (21)	53 (38)	85 (62)

^a^Based on Chi-square test, ^b^*p* < 0.05.
